# The feasibility of a multi-site, clinic-supported, and tailored neuro-oncology exercise program

**DOI:** 10.1093/nop/npae093

**Published:** 2024-10-10

**Authors:** Julia T Daun, Lauren C Capozzi, Tana Dhruva, Gloria Roldan Urgoiti, Meghan H McDonough, Emma McLaughlin, Mannat Bansal, Allan Brett, Jacob C Easaw, Margaret L McNeely, George J Francis, Tanya Williamson, Jessica Danyluk, Paula A Ospina, Christine Lesiuk, Paula de Robles, Catriona Leckie, S Nicole Culos-Reed

**Affiliations:** Faculty of Kinesiology, University of Calgary, Calgary, Alberta, Canada; Faculty of Kinesiology, University of Calgary, Calgary, Alberta, Canada; Department of Clinical Neurosciences, Cumming School of Medicine, University of Calgary, Alberta, Canada; Faculty of Kinesiology, University of Calgary, Calgary, Alberta, Canada; Department of Medical Oncology, Tom Baker Cancer Centre, Alberta Health Services, Calgary, Alberta, Canada; Faculty of Kinesiology, University of Calgary, Calgary, Alberta, Canada; Faculty of Kinesiology, University of Calgary, Calgary, Alberta, Canada; Faculty of Kinesiology, University of Calgary, Calgary, Alberta, Canada; Faculty of Kinesiology, University of Calgary, Calgary, Alberta, Canada; Department of Medical Oncology, Cross Cancer Institute, Edmonton, Alberta, Canada; Department of Physical Therapy, University of Alberta, Edmonton, Alberta, Canada; Department of Oncology, Cancer Care Alberta, Edmonton, Alberta, Canada; Department of Clinical Neurosciences, Cumming School of Medicine, University of Calgary, Alberta, Canada; Department of Oncology, Cumming School of Medicine, University of Calgary, Calgary, Alberta, Canada; Faculty of Kinesiology, University of Calgary, Calgary, Alberta, Canada; Faculty of Kinesiology, University of Calgary, Calgary, Alberta, Canada; Department of Physical Therapy, University of Alberta, Edmonton, Alberta, Canada; Department of Medical Oncology, Tom Baker Cancer Centre, Alberta Health Services, Calgary, Alberta, Canada; Department of Medical Oncology, Tom Baker Cancer Centre, Alberta Health Services, Calgary, Alberta, Canada; Department of Medical Oncology, Tom Baker Cancer Centre, Alberta Health Services, Calgary, Alberta, Canada; Faculty of Kinesiology, University of Calgary, Calgary, Alberta, Canada; Department of Oncology, Cumming School of Medicine, University of Calgary, Calgary, Alberta, Canada; Department of Psychosocial Resources, Tom Baker Cancer Centre, Alberta Health Services, Alberta, Canada

**Keywords:** exercise, exercise oncology, feasibility, implementation, neuro-oncology

## Abstract

**Background:**

To address the lack of access to supportive cancer care resources, the purpose of this study was to examine the feasibility of a tailored exercise program for neuro-oncology patients.

**Methods:**

Patients with a primary brain tumor diagnosis, >18 years, and able to consent in English were recruited at 2 tertiary cancer centers in Alberta. Recruitment occurred via the electronic medical record as well as self-referral. A 12-week, tailored exercise intervention with health coaching was delivered in both one-on-one and group-based formats, either in-person or online. Measures of feasibility included tracking referral, enrollment, intervention completion and adherence, measurement completion, fidelity, participant satisfaction, and safety. Participant-reported outcomes and functional fitness were assessed at baseline and 12 weeks. Objective physical activity was tracked via a Garmin activity tracker.

**Results:**

Recruitment occurred between April 2021–December 2022. *N* = 70 patients enrolled in the study and *n* = 51 completed the intervention. The referral rate was 31%, the enrollment rate was 66%, and intervention completion and adherence rates were 82.3% and 89.7%. At baseline and 12 weeks, measurement completion rates were 100% and 77.4% for patient-reported outcomes, and 98.4% and 75.8% for functional fitness. The average wear-time for the activity tracker was 72.8%. Fidelity of intervention delivery was 100% for exercise sessions and 87.8% for health coaching. Overall participant satisfaction was 86.5%. No major and 4 minor adverse events occurred.

**Conclusions:**

Delivery of a tailored neuro-oncology exercise program with referral included via the electronic medical record is feasible. Future work is needed to optimize tailored programming as well as to address factors critical for implementation into standard cancer care.

**Clinical Trials Registration:**

NCT04831190 (https://clinicaltrials.gov/ct2/show/NCT04831190).

## Graphical Abstract

**Graphical Abstract:**
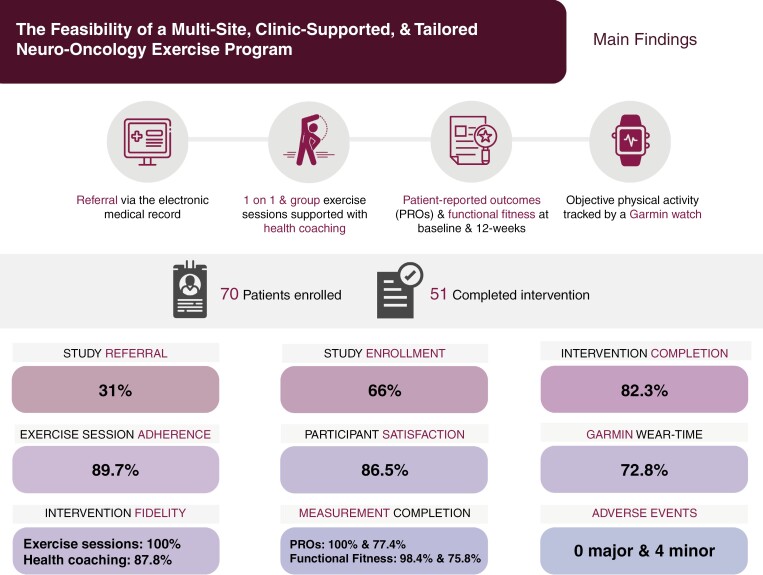

**Key Points**

• Delivery of a tailored neuro-oncology exercise program with referral included via the electronic medical record is feasible.
• Future work is needed to optimize tailored programming and address factors critical for implementation into neuro-oncology care.

**Importance of the Study**

Neuro-oncology patients require a tailored and flexible approach to support exercise participation. Establishing the feasibility of delivering a tailored exercise program across neuro-oncology care is a critical first step to successful implementation. To the best of our knowledge, ACE-Neuro is the largest neuro-oncology exercise intervention to date delivered to 2 tertiary cancer centers. Notably, this work has built the groundwork for establishing direct referral through the electronic medical record and building exercise resources into standard neuro-oncology cancer care.

Evidence supporting the safety and efficacy of exercise across the cancer care continuum for all individuals living with cancer is strong.^1–3^ Substantive research in the field of exercise oncology has led to the development of exercise oncology-specific guidelines as well as pathways to streamline access from clinics to community-based exercise programming.^4,5^ While these findings may support understanding general exercise prescription needs for individuals living with and beyond cancer, evidence is still growing in complex and clinically underserved tumor groups. Neuro-oncology patients represent one of these complex and underserved groups in exercise oncology research.^6^

The incidence of primary brain tumors has increased over the last few decades, with the latest statistics estimating 330 000 global diagnoses each year.^7^ Neuro-oncology patients often undergo multimodal and intensive treatments (ie, surgery, chemotherapy, radiotherapy, targeted therapy, prolonged use of steroids, and other medications)^8,9^ that lead to both functional (eg, physical function, cognition) and psychosocial (eg, quality of life, depression) challenges.^6,10–13^ Further, neuro-oncology patients are often presented with poor survival prognoses that can lead to additional psychological, financial, and end-of-life distress, impacting both patients and their families.^14^ Given the nature of primary brain tumors, addressing treatment-related side effects and functional and psychosocial outcomes while living with brain cancer is a growing need. Specifically, including supportive cancer care resources to address the pillars of wellness for these individuals is critical. Exercise is one such resource that can address the functional and psychosocial needs of the neuro-oncology patient population.^6,15,16^

The exercise research and available resources in neuro-oncology are limited, with evidence to date concluding that it is safe and feasible to recruit this patient population,^17,18^ assess patient outcomes (eg, functional and psychosocial outcomes^17–19^), as well as conduct interventional research.^19–26^ Furthermore, evidence from a 2021 systematic review has shown that exercise is potentially beneficial to this population for outcomes such as symptom severity, functional fitness, cognition, and quality of life.^27^ Notwithstanding this preliminary work, we have yet to examine streamlining access to exercise resources, providing tailored exercise programming, and implementing exercise into standard neuro-oncology care. There is thus a critical need to assess the feasibility of exercise implementation across the neuro-oncology clinical timeline.

Understanding referral, recruitment, and implementation of a tailored exercise program for neuro-oncology patients within the North American neuro-oncology setting will (1) help close the research-to-practice gap,^28^ (2) address integral uncertainties related to clinical logistics to ensure the development and delivery of a clinic-supported program, and (3) understand participant experiences across the study process to inform patient-centered care.^29^ The purpose of this study was thus to examine the feasibility of a clinic-supported, tailored exercise program for neuro-oncology patients—the Alberta Cancer Exercise-Neuro (ie, ACE-Neuro) study within an effectiveness-implementation framework.^30^

## Methods

### Procedure, Participants, and Recruitment

This study was approved by the University of Calgary Health Research Ethics Board of Alberta (HREBA)—Cancer Committee (CC; HREBA. CC-20-0322). The complete study protocol is published separately.^31^ Briefly, the ACE-Neuro study recruited neuro-oncology patients from 2 clinical sites across Alberta—Calgary (Tom Baker Cancer Center) and Edmonton (Cross Cancer Institute). Eligibility for the study included: a diagnosis of a primary brain tumor, being pre-, on, or completed treatment and over 18 years of age, and the ability to consent in English. Patient referral occurred either via the electronic medical record (Calgary) or via self-referral (ie, contacting the study team after getting information from a study brochure, poster, or word of mouth; Calgary and Edmonton). In Calgary, once a patient was referred, a Neuro-Oncology Rehabilitation Triage Clinic^32^ triaged patients to either physiotherapy, occupational therapy, physiatry, exercise, or a combination of services to support access to wellness resources. The triage clinic assessed patients’ functional and cognitive status and determined referral to exercise based on no new/unmanaged medical conditions, as well as scores of ≥5 on the Short Physical Performance Battery protocol, <3 for ECOG (Eastern Cooperative Oncology Group), and >50 for KPS (Karnofsky Performance Status).^32^ In Edmonton, patients were initially meant to be referred via their usual occupational therapy assessment at the Cross Cancer Institute, but due to personnel changes at the start of recruitment, this was no longer an option. Thus, patients could only self-refer in Edmonton. To support participant safety and appropriate tailoring of the exercise intervention, pre-exercise screening procedures occurred across both clinical sites and included collecting clinical and sociodemographic characteristics, health history (ie, cancer history, other medical conditions), and exercise history and preferences.

### Exercise Intervention

The ACE-Neuro exercise intervention included 12 weeks of supervised and tailored one-on-one and group-based sessions, embedded within a positive motivational framework (ie, “*Exercise and Educate*”—the delivery of education and behavior change support during exercise sessions). Sessions were delivered by qualified exercise professionals with specific cancer and exercise training. Six qualified exercise professionals from exercise oncology research groups in Calgary and Edmonton delivered (ie, led sessions) or supported delivery (ie, assisted with sessions) of the intervention, 4 of which were CSEP-Clinical Exercise Physiologists. Sessions were delivered based on participant preference—either online (ie, via ZOOM) or in-person (ie, at a cancer exercise facility). Due to the COVID-19 pandemic, the first 12 months of intervention delivery in Calgary only occurred via ZOOM. In Edmonton, in-person intervention delivery occurred from the start to the end of recruitment. All participants began with one-on-one sessions for at least 2 weeks and then had the option to join group-based sessions. Based on the clinical judgment of the exercise professional or participant preference, sessions could continue to be delivered one-on-one for the 12 weeks.

Exercise programs were tailored to meet individual needs, followed principles of *Frequency, Intensity, Time, and Type* (FITT), and included multimodal prescriptions of aerobic, resistance, balance, and flexibility training.^31^ Prescriptions were based on established cancer and exercise guidelines^1^ as well as brain tumor type, treatment type, place on the cancer continuum, and individual factors (eg, exercise preferences).^31^ Exercise sessions were designed to be delivered twice weekly (ie, frequency). Exercise intensity was based on acute self-reported levels of energy, and fatigue and was monitored using Borg’s Rating of Perceived Exertion (RPE; 1–10) scale.^33^ Session time (ie, length) was designed for 30–60 minutes with a 5–10-minute warm-up (RPE ~1–3), a 15–40-minute main circuit (RPE ~2–6), and a 10–15-minute cool down (RPE ~1–2).^31^ Qualified exercise professionals utilized established group program templates (see Appendix A) to then modify each participant’s tailored program (see sample in Appendix B). Group sessions were delivered in a rotating format (ie, week 1: program A, week 2: program B, week 3: program C). Qualified exercise professionals spent approximately 30-45 minutes preparing each tailored program and 15 minutes preparing for and charting post each one-on-one and group-based session (ie, time commitment of 90 minutes per session). To support exercise behavior change and wellness, participants were offered weekly 15–30-minute health coaching sessions as part of the intervention, either during or outside of the one-on-one or group-based sessions. Health coaching was delivered by exercise professionals with health behavior change training and followed a standardized protocol that addressed key health coaching principles of: (1) ensuring a participant-centered approach, (2) self-determining goals, (3) supporting the self-discovery process, (4) encouraging participant accountability, (5) providing education, (6) summarizing and motivating, and (7) building coach-participant rapport.^34^

### Measures

#### Feasibility rates.—

To address the primary outcome of feasibility, pre-determined feasibility rates were established for referral (≥50%; the number of patients referred from the neuro-oncology clinic to the research team), enrollment into the study (≥50%; the number of patients who consented after hearing the full study introduction), intervention completion and adherence (≥50%; completion of full intervention and adherence to each exercise session), measurement completion at baseline and 12 weeks (≥60%; completion of patient-reported outcomes and functional fitness measures), and no major adverse events. Adverse events were tracked using a standardized University of Calgary reporting system that classifies adverse events as 1 of the 3 levels.^31^ Level 1 is classified as a minor incident with no lost time beyond the day of injury (ie, temporary, immediate care); level 2 is classified as needing medical aid with no lost time beyond the day of injury (ie, medical care beyond first aid); and level 3 is classified as serious injury or death (ie, a major adverse event).

#### Intervention fidelity.—

Fidelity was assessed for intervention, assessment, and health coaching delivery via standardized fidelity tracking checklists and session recordings for health coaching. Tracking of fidelity was added to the ACE-Neuro protocol after the protocol paper was published.^31^ First, exercise specialists utilized a tracking checklist to document each exercise session prescription (FITT) as well as fatigue and energy pre- and post-exercise sessions. Specifically, tracking included session frequency, intensity (ie, participant-reported exertion via the RPE Scale^33^), time (ie, session duration and time spent on each component of the multimodal program), type of exercise performed (ie, aerobic, resistance, balance, and flexibility), the format of delivery (ie, in-person or online; one-on-one or group), any deviations from the protocol, and any additional participant-related concerns (eg, treatment-related side effects). The lead author (JTD) evaluated all tracking checklists for fidelity. Second, health coaching tracking checklists documented session length, format (ie, one-on-one or group; in-person, via ZOOM, or via phone call), and topics covered. Ten percent of health coaching sessions were recorded at random and assessed for fidelity by 2 independent evaluators (JTD and MB) using a checklist that compared session delivery to the intended protocol. Third, to prevent bias across assessments of functional fitness, interventionists differed from assessors and the same assessors were protocolized to conduct pre-to post-assessments. Finally, as an effectiveness-implementation study,^30^ of the study team met on a bi-weekly basis to discuss quality improvement of intervention delivery. All changes to study protocols were tracked.

#### Measurement completion.—

Measurement completion pertained to patient-reported outcomes (ie, quality of life, cognition, fatigue, physical activity levels, and symptom burden) and functional fitness (ie, body composition, muscular strength, muscular endurance, balance, flexibility, and cardiorespiratory fitness) assessed at baseline and 12-weeks. Patient-reported outcomes were completed online, via REDCap^35^ and functional fitness was assessed either online, via ZOOM, or in-person (based on participant preference) and followed the same protocol regardless of location. Each functional fitness assessment was 20–45 minutes in duration. Objective physical activity was tracked via the use of the Vivosmart 4 Garmin Activity Tracker across intervention delivery. Participants were instructed to wear the activity tracker for a minimum of ten hours per day. Reasons for not wearing the tracker were tracked. See the full description of participant outcome measures in the ACE-Neuro protocol paper.^31^ Participant outcome results will be reported separately.

#### Participant satisfaction.—

Participant satisfaction with the study was measured via a post-intervention satisfaction questionnaire that included questions on (1) satisfaction with the study staff, (2) satisfaction with the exercise program, (3) acceptability of assessments, (4) intentions to continue exercising over the next year, and (5) perceptions of study benefits to self and others.

### Analysis

Analysis of feasibility included descriptive statistics for participant characteristics, feasibility measures, adverse events, fidelity, objective physical activity use, and participant satisfaction from the post-intervention survey, and are presented as mean ± standard deviation or percentages.

## Results

### Participant Characteristics

Participant demographics are presented in Table 1 and clinical characteristics are presented in Table 2. The mean age of participants was 49.5 ± 12.4 years, 51.6% of participants self-identified as male, and over half completed University/College-level education (*n* = 33, 53.2%). Most participants self-identified as White (*n* = 48, 77.4%), were diagnosed with glioblastoma (*n* = 22, 35.5%), and were post-treatment (*n* = 40, 64.5%). The average time since diagnosis was 61.5 ± 79.5 months, with the most common (mode) 11 months, and the median reported as 24 months. All clinical characteristics have been verified by health record check.

**Table 1.** Participant Demographics, *n* = 62

Demographic variable	Number of participants
Self-identified sex	
Male	32
Female	30
Age: mean ± SD, years	49.5 ± 12.4 (range: 29–81)
Marital status	
Never married	6
Married	47
Common law	2
Separated	1
Divorced	6
Education	
Some High School	4
Completed High School	5
Some University/College	5
Completed University/College	33
Some Graduate School	3
Completed Graduate School	12
Annual family income, CDN$	
<$20 000	7
$20 000–$39 999	6
$40 000–$59 999	6
$60 000–$79 999	7
$80 000–$99 999	4
>$100 000	16
Prefer not to answer	16
Employment status	
Short-term disability	2
Long-term disability	27
Retired	17
Part-time	1
Homemaker	3
Full-time	4
Unemployed	6
Other	2
Self-identified racial background^*^	
Black	2
East Asian	2
Indigenous	2
South Asian	3
Southeast Asian	3
White	48
White and South Asian	1
Not specified	1

^*^Reported based on the Canadian Institute for Health Information’s Guidance on the Use of Standards for Race-Based and Indigenous Identity Data Collection and Health Reporting in Canada.^36^

**Table 2.** Clinical Characteristics, *n* = 62

Clinical characteristic	Number of participants
Time since diagnosis: mean ± SD, months	61.5 ± 79.5
Type of primary brain tumor	
Glioblastoma	22
Oligodendroglioma	15
Astrocytoma	12
Meningioma	4
Germinoma	2
Medulloblastoma	1
Presumed glioma	1
Malignant glioma not otherwise specified	1
Craniopharyngioma	1
Pineocytoma	1
Subependymoma	1
Pharyngeal cranial cyst	1
Histologic grade	
1	3
2	11
3	15
4	26
Unknown	7
Treatment status	
Pretreatment^*^	1
On treatment	21
Off treatment	40
Treatment type	
Surgery alone	1
Surgery + radiation	6
Surgery + chemoradiation	2
Surgery + radiation + adjuvant chemotherapy	14
Surgery + chemoradiation + adjuvant chemotherapy	37
Surgery + chemoradiation + adjuvant chemotherapy + proteasome inhibition^**^	1
Chemoradiation + adjuvant chemotherapy	1
Smoking status	
Never smoked	40
Ex-smoker	19
Occasional smoker	1
Regular smoker	2
Alcohol drinking status	
Never drinker	12
Ex-drinker	16
Occasional drinker	23
Social drinker	9
Regular drinker	2

^*^Pretreatment is before surgery, chemotherapy, and/or radiation.

^**^Proteasome Inhibition was part of a clinical trial.

### Feasibility Results

#### Referral and enrollment.—

**Figure 1.**
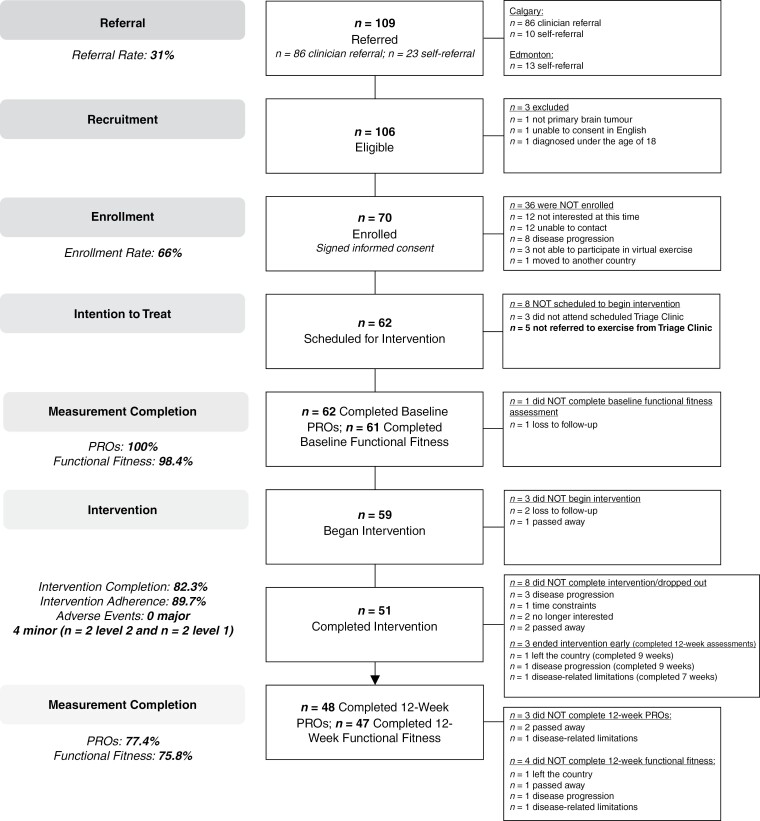
Participant flow and key feasibility statistics.

The ACE-Neuro study recruitment flow chart can be found in Figure 1. Recruitment in Calgary was open for 20 months between April 2021 and December 2022. Recruitment in Edmonton was open for 11 months between January and December 2022. In Calgary, approximately 280 patients were seen in the neuro-oncology clinic during study recruitment (ie, 14 patients per month), of which *n* = 86 patients were referred to the study for a referral rate of 31% (ie, via the electronic medical record). Ten patients either self-referred (ie, via study brochure; *n* = 3) or were referred through the Health and Wellness Lab (ie, via another study or colleague; *n* = 7). Complete reasons for not referring via the electronic medical record were not collected for every patient seen during the recruitment period. However, clinical members indicated reasons they did not refer included: (1) competing priorities in the clinic and forgetting to refer, (2) patients’ lack of interest, and (3) clinical judgment that a patient would not be eligible or appropriate for the study (eg, those at end of life). Of the 96 total referrals in Calgary, 93 patients were eligible for the study. Reasons for exclusion included: (1) not being diagnosed with a primary brain tumor (*n* = 1), (2) unable to consent in English (*n* = 1), and (3) being diagnosed under the age of 18 (*n* = 1). In Edmonton, 13 patients self-referred and enrolled in the study. Across both clinical sites, *n* = 70 of 106 total referred and eligible patients enrolled in the study for an enrollment rate of 66%. Reasons for not enrolling (Calgary site; *n* = 36) included: (1) not being interested (*n* = 12), (2) unable to contact (*n* = 12), (3) disease progression (*n* = 8), (4) unable to participate in virtual exercise (*n* = 3), and (5) moved to another country (*n* = 1). Of the 70 enrolled patients, 8 did not begin the intervention either because they (1) did not attend their scheduled triage clinic appointment (*n* = 3) or (2) were not referred to exercise from the triage clinic (*n* = 5). Sixty-two patients were thus scheduled to begin the exercise intervention and are included in the calculation of measurement and intervention completion rates. Of these *n* = 62 participants, *n* = 59 began the exercise intervention. Reasons for not beginning the exercise intervention included: death (*n* = 1) and loss of follow-up (*n* = 2; Figure 1).

#### Intervention completion and adherence.—

Of the *n* = 59 participants who began the exercise intervention, *n* = 48 completed the full 12 weeks, while *n* = 3 completed part of the intervention, with early completion (range of 7–9 weeks total). Reasons for early completion included disease progression (*n* = 1), leaving the country (*n* = 1), or disease-related limitations (*n* = 1). Eight participants withdrew from the study due to disease progression (*n* = 3), death (*n* = 2), time constraints (*n* = 1), and not being interested (*n* = 2). The total number of participants who completed the intervention (full or partial) was *n* = 51 (intervention completion rate of 82.3%) and are thus included in the presentation of intervention adherence, fidelity, and participant satisfaction results. The adherence rate for the exercise intervention (ie, the exercise sessions) was 89.7% ± 15.2 (range 46.1%–100%). Four participants had an adherence rate below 60% for reasons including: (1) transportation challenges, (2) time constraints, (3) health issues (eg, illness), and (4) treatment-related side effects (eg, fatigue).

One-on-one health coaching was delivered to 47 of the 51 participants (92.2%) with an average of 5.6 ± 5 sessions per participant over the 12 weeks and an average duration of 20.1 ± 18.5 minutes per session (range 5–60 minutes). Four participants did not receive one-on-one formal health coaching due to not being interested and felt that the “*Exercise and Educate*” format of exercise sessions was sufficient to support their adherence to and maintenance of exercise. Of the 29 participants who attended group exercise sessions, 26 (89.7%) participated in group health coaching (range of 1–5 participants per session) with an average of 15.8 ± 6.2 minutes in duration per session. Topics covered in health coaching sessions included goal setting, stress management, overcoming barriers and facilitators, self-monitoring, self-compassion, adjusting to changes, social support, and sleep.

#### Fidelity.—

Fidelity tracking checklists were completed for 96% of one-on-one and 99.9% of group exercise sessions. All exercise sessions (100%) followed the intended protocol. Fidelity for the tracking assessments pre-exercise sessions was 96.7% for fatigue and 99.5% for energy for one-on-one sessions, and 100% for both tracking measures in group sessions. Fidelity for tracking assessments post-exercise sessions was 96% for fatigue and 96.3% for energy for one-on-one sessions, and 99.5% (fatigue) and 100% (energy) for group sessions. Across one-on-one sessions, tracking participant-reported RPE was collected following only the main circuit portion of exercise sessions for the first 29 participants (ie, “initial tracking”) and following the warm-up, main circuit, and cool-down for 22 participants (ie, “revised tracking”). This change was made after one of the study team’s bi-weekly meetings for quality improvement of intervention delivery. The fidelity of tracking RPE following only the main circuit (ie, first 29 participants) was 97%. The fidelity of tracking RPE for the remaining 22 participants was 93.3% after the warm-up, 94.2% after the main circuit, and 85.9% after the cool-down. Within group sessions, RPE was collected following only the main circuit portion of exercise sessions for 19 participants (ie, “initial tracking”) and following the warm-up, main circuit, and cool-down for 8 participants (ie, “revised tracking”). The fidelity of tracking RPE across both methods in group sessions was 100%.

**Figure 2.**
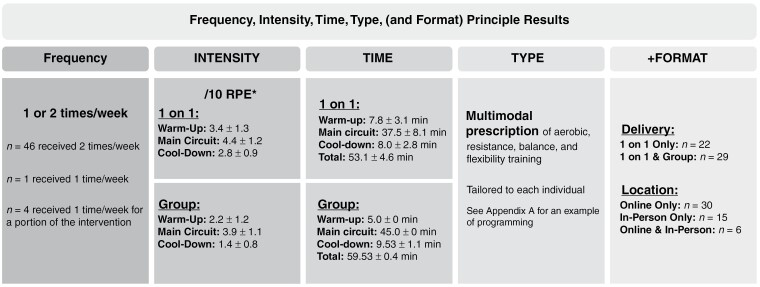
The ACE-neuro exercise prescription: frequency, intensity, time, type (and format) results. Based on the *n* = 51 that completed the intervention. *Rating of Perceived Effort/Exertion (RPE). Self-reported by participants using the Borg Perceived Exertion Scale (1998). For one-on-one sessions, RPE was collected only for the main circuit for 29 participants and for 22 participants for each of the warm-up, main circuit, and cool-down components of sessions. For group sessions, RPE was collected only for the main circuit for 19 participants and for 8 participants for each of the warm-up, main circuit, and cool-down components of sessions.

All participants received the intended multimodal exercise prescriptions of aerobic, resistance, balance, and flexibility training.^31^ The intervention frequency, intensity, time, type, *and format* (FITT + F) principle is presented in Figure 2. The frequency of intervention delivery was 1 or 2 times per week. A total of *n* = 46 participants received sessions twice weekly and *n* = 1 participant received sessions once weekly. Four participants received once weekly for a portion of the intervention (range of 4–5 weeks) due to time constraints (*n* = 1), transport limitations (*n* = 2), and disease progression (*n* = 1). Across one-on-one sessions, participant-reported intensity (out of 10)^33^ was 3.4 ± 1.3 for warm-up, 4.4 ± 1.2 for the main circuit, and 2.8 ± 0.9 for the cool-down. Across group sessions, intensity was reported as 2.2 ± 1.2 for warm-up, 3.9 ± 1.1 for the main circuit, and 1.4 ± 0.8 for the cool-down. The mean session time was 53.1 ± 4.6 minutes for one-on-one sessions and 59.53 ± 0.4 minutes for group sessions. Across intervention delivery, *n* = 30 participants participated in online sessions only, *n* = 6 in both online and in-person, and *n* = 15 in in-person only. Twenty-two participants received one-on-one sessions only (ie, due to higher needs and/or participant preference) and *n* = 29 participants received both one-on-one and group-based sessions.

The fidelity of health coaching was 87.8% for both one-on-one and group-based sessions. The health coaching principle of “summarizing” had the lowest score within group-based sessions. The fidelity of assessments of functional fitness was 97.9%, wherein 47/48 of baseline and 12-week assessments were conducted by the same assessor at both timepoints.

#### Measurement completion.—

**Figure 3.**
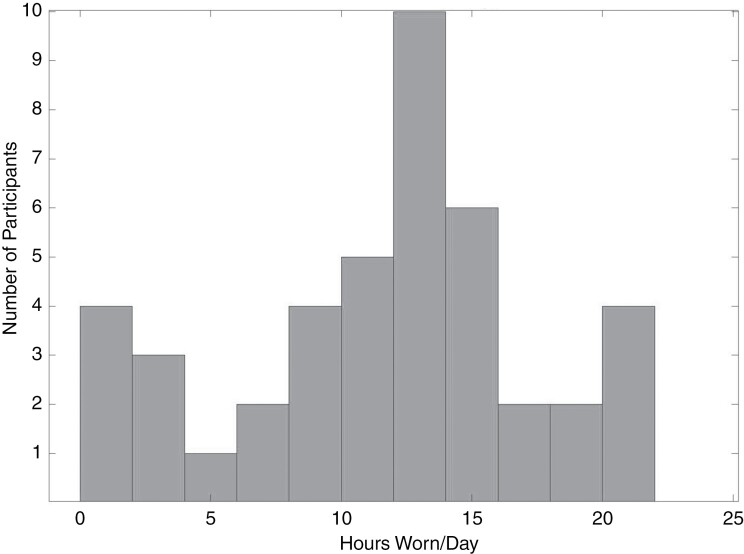
Distribution of average hours worn per day across participants. Based on the *n* = 51 who completed the intervention.

Measurement completion rates were 100% and 77.4% for baseline and 12-week patient-reported outcomes, and 98.4% and 75.8% for baseline and 12-week assessments of functional fitness (Figure 1). With regards to the measurement of objective physical activity levels, 8 of the 51 participants who completed the intervention did not wear the activity tracker at all. Reasons for not wearing the tracker included disease-related limitations (*n* = 3), technology limitations (*n* = 2), lack of interest (*n* = 2), and added stress (*n* = 1). Of the 44 participants who did wear the activity tracker, it was worn for an average of 11.5 ± 5.78 hours per day (Figure 3) and for an average of 72.8% of the 12-week intervention length (range of 21.7%–100%). Forty-three of the fifty-one wore the activity tracker for more than 60% of the intervention. The 8 participants who wore the watch less than 60% of the time reported barriers such as lack of interest (*n* = 4), technology issues (*n* = 2), disease progression (*n* = 1), and disease-related limitations (*n* = 1).

#### Adverse events.—

Adverse events were tracked for all enrolled participants (ie, those who signed informed consent; *n* = 70). Across the study period, 2 level 2 adverse events (ie, needing medical aid with no lost time beyond the day of injury) were reported during one-on-one exercise sessions, including a seizure and an episode of pre-syncope. In both cases, adverse events were discussed by the research team, reported to the ethics board, and brought to the attention of the participants’ medical oncologists. Both participants were deemed safe to continue exercise and returned to their exercise sessions without delay or disruption to the protocol. Four level-one adverse events (ie, minor incidents with no lost time beyond the day of injury) were reported by participants to the study team for events that took place outside of exercise sessions. These included (1) an episode of syncope and a subsequent fall, (2) skin irritation, (3) musculoskeletal pain, and (4) a headache. These events were discussed with the participant and reported to the ACE-Neuro study coordinator. No major adverse events occurred (ie, no serious injury or death).

#### Participant satisfaction.—

Of the *n* = 51 participants who completed the intervention, *n* = 41 (80.4%) completed the post-study satisfaction survey. Table 3 presents the survey results. Overall, participants reported feeling satisfied with the study staff (91.5%), the exercise program (85.6%), and attending and completing assessments (83.3%). Intention to continue exercising in the next year was 80.3% across all participants and the average perception of study benefit was 91.7%. The overall participant satisfaction with the study was 86.5%.

**Table 3.** Results From the ACE-Neuro Satisfaction Survey

Satisfaction with the ACE-Neuro Staff	91.5%
Satisfaction with the ACE-neuro exercise program	85.6%
Intentions to continue exercising in the next year	80.3%
Acceptability of assessments and travel to the exercise facility	83.3%
Perceptions of study benefits to self and others	91.7%
***Overall satisfaction with participation in ACE-neuro***	***86.5%***

*Satisfaction was measured on a 0–100 scale.*

## Discussion

The purpose of this study was to examine the feasibility of ACE-Neuro—a clinic-supported, tailored exercise program for neuro-oncology patients. ACE-Neuro was developed to support “*real-world*” delivery and implementation into standard neuro-oncology care. To the best of our knowledge, ACE-Neuro is the largest multi-site neuro-oncology intervention to date, with *n* = 51 completing the intervention. Much of the interventional research conducted in neuro-oncology to date has included small sample sizes *n* = 10–30 participants.^19,20,22–26^ Moreover, this is the first exercise oncology study at our local cancer center in Calgary (ie, Tom Baker Cancer Center) to have a referral option within the electronic medical record.

Overall, ACE-Neuro exceeded the pre-determined criteria for all feasibility measures other than referral rate (ie, the number of patients referred from the neuro-oncology clinic to the research team). Enrollment was high, measurement completion was good, intervention completion and adherence, fidelity of delivery, and participant satisfaction were excellent, and there were no major adverse events. Tailoring exercise prescriptions, the flexibility of intervention delivery (eg, timing, format of delivery), and ongoing communication with the clinical team were instrumental in supporting the feasibility of ACE-Neuro.

Though the 31% referral rate did not meet our minimum threshold of ≥50%, this is a conservative estimate based on all patients seen in the neuro-oncology clinic, some of whom may not have been eligible for the study given the advanced nature of brain tumors. Nevertheless, the low referral rate may speak to the need to support clinicians’ capabilities (eg, knowledge of exercise oncology), opportunities (eg, suitable referral and screening pathways), and motivation (eg, support from leadership, adjustments to clinical workflow) to refer patients to appropriate rehabilitation and exercise resources.^37^ Previous studies have advocated for the role of clinicians as critical for informing patients about available programming,^4,38–40^ with more engaged and knowledgeable clinicians resulting in increased referral rates to appropriate resources.^41^ However, it is evident from this work and others^40^ that lack of time and limited referral pathways remain barriers to referral. A way forward is working with clinical teams to identify what strategies can best support their roles in advocating for and referring to exercise oncology programming. In our ongoing work, we are utilizing the *Cancer Rehabilitation and Exercise Screening Tool*^32^ to improve in-clinic screening, support referral, and ensure appropriate triage to the “*right*” exercise resources.

In comparing the 2 neuro-oncology clinical sites, Calgary’s recruitment period was 9 months longer and enrolled 57 more eligible patients than Edmonton’s, where recruitment was dependent solely on self-referral. Though a longer recruitment period occurred, and 194 potentially eligible patients were not referred in Calgary, the clinician referral still facilitated more enrollments than self-referral alone, pointing to the value of integrating referral via the electronic medical record to support patient access to exercise programming. Furthermore, neuro-oncology exercise intervention studies to date have reported enrollment rates of 25%–100%, placing ACE-Neuro at 66% and on the higher end of enrollment feasibility.

The ACE-Neuro intervention adherence rate of 89.7% is comparable to other exercise studies delivered to neuro-oncology patients (range of 61.1%–100%).^19,20,22–26^ For example, Gehring and colleagues delivered an at-home, unsupervised, 6-month aerobic intervention with remote guidance and a mean adherence rate of 79%.^19^ Capozzi and colleagues reported an adherence rate of 61.1% for an in-person, supervised, group-based 12-week multimodal intervention.^20^ Adherence rates of 90%–100% have been reported across shorter interventions, including 2- or 3-times weekly supervised yoga sessions to both patients undergoing radiation therapy and their caregivers^23,24^ and a supervised 6-week in-patient-out-patient individualized aerobic training program.^22^ Nowak and colleagues also reported a 90% adherence rate for a supervised group-based multimodal exercise program for the duration of chemoradiation.^26^

The feasibility of health coaching in ACE-Neuro (delivered to 92.2% of participants) points to the potential role of behavior change counseling in supporting adherence to and maintenance of exercise, especially for a patient population with a high symptom burden.^34,42,43^ Given the feasibility and these potential benefits, future work should examine the inclusion of tailored behavior change strategies delivered via a health coaching framework to facilitate exercise adherence and longer-term maintenance. Though the fidelity of health coaching delivery was high (87.8%) across both one-on-one and group-based sessions, ‘summarizing’ within group-based sessions was often missing. This speaks to the need to ensure health coaches have the training and resources (eg, checklists) to support their effective delivery of key health coaching principles in each session. Finally, our detailed tracking of the FITT(+F) principle, with participant-reported values for intervention intensity (ie, rating of perceived exertion) and participant-centered decisions for all components of prescription, provides valuable information on what is a feasible exercise prescription for neuro-oncology patients.

## Limitations and Future Directions

Though the present work was conducted in a clinical setting to support implementation and sustainability, it is important to consider that the context in which this was done may be different from other clinical settings. We had the resources and clinical buy-in to prioritize enhanced referral pathways, however, this may not be possible in all settings, and in particular lower-income countries may not have the necessary infrastructure to mimic our pathway. Second, while cost analyses are ongoing, it is clear that the cost to deliver one-on-one sessions is higher than group-based programming. Without funding support, one-on-one delivery, though warranted for the neuro-oncology patient population, becomes more inaccessible. Third, although ACE-Neuro was inclusive for brain tumor type, treatment type, and place on the cancer continuum, as well as increased access for participation via in-person and remote options (eg, to reach those living outside of the major urban settings where in-person classes were offered), it is not inclusive of all populations, including any non-English participants. Future research, funding, and trained personnel are needed to provide equitable access and opportunities for engagement in exercise oncology programming for non-English speaking patients.^44^ Finally, our sample size limits the ability to conduct sub-analyses. Based on our preliminary work, disease progression may be associated with decreased adherence to programming. Ongoing work with larger sample sizes will allow us to elucidate this and other (eg, baseline physical activity levels) potential relationship.

## Conclusion

The delivery of a tailored 12-week multimodal exercise intervention for neuro-oncology patients is feasible. There is a need to identify strategies for supporting referrals by addressing clinical team members’ capabilities, opportunities, and motivations to refer. In addition, ongoing work is exploring how exercise may be used as a supportive cancer care resource to help manage treatment-related side effects and improve functional and psychosocial outcomes in neuro-oncology. Exercise has a promising role in standard neuro-oncology care and with continued implementation efforts, evidence can inform practice to enhance patients’ well-being and overall quality of life.

## Supplementary Material

npae093_suppl_Supplementary_Appendix_A

npae093_suppl_Supplementary_Appendix_B

## Data Availability

Data will be made available on request.
