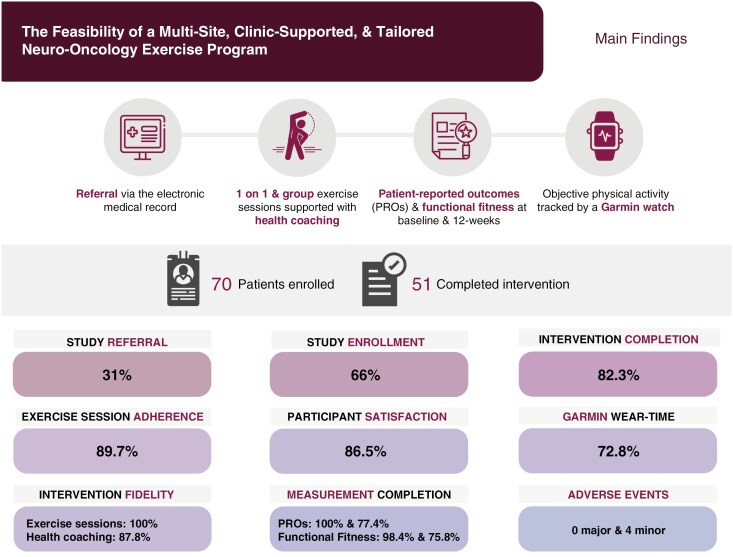# Corrigendum to: The feasibility of a multi-site, clinic-supported, and tailored neuro-oncology exercise program

**DOI:** 10.1093/nop/npaf017

**Published:** 2025-02-12

This is a correction to: Julia T Daun, Lauren C Capozzi, Tana Dhruva, Gloria Roldan Urgoiti, Meghan H McDonough, Emma McLaughlin, Mannat Bansal, Allan Brett, Jacob C Easaw, Margaret L McNeely, George J Francis, Tanya Williamson, Jessica Danyluk, Paula A Ospina, Christine Lesiuk, Paula de Robles, Catriona Leckie, S Nicole Culos-Reed, The feasibility of a multi-site, clinic-supported, and tailored neuro-oncology exercise program, *Neuro-Oncology Practice*, 2024; https://doi.org/10.1093/nop/npae093

The following changes have been made to the originally published manuscript. The graphical abstract has been changed. The completion rates for patient-reported outcomes (PROs) and functional fitness were reversed within the original graphical abstract. This is replaced in the article with the revised version presents the correct completion rate results: 100% (baseline) and 77.4% (12-weeks) for PROs and 98.4% (baseline) and 75.8% (12-weeks) for functional fitness.

There was also an error in Figure 1. In the penultimate box of the third column there was an errored repeated heading. The second heading in the box has been emended to read: “”n=3 ended intervention early (completed 12-week assessments)” instead of the duplicated: “n=8 did NOT complete intervention/dropped out”.

The emendations have been made to the article.