# Supplementary material for: The feasibility of a multi-site, clinic-supported, and tailored neuro-oncology exercise program
Source: Neurooncol Pract. 2024 Oct 10;12(1):131–42. doi: 10.1093/nop/npae093 (PMC11798609; doi:10.1093/nop/npae093)
Supplement: npae093_suppl_Supplementary_Appendix_A [file npae093_suppl_supplementary_appendix_a.pdf]

UNIVERSITY OF CALGARY
FACULTY OF KINESIOLOGY
Health and Wellness Lab

UNIVERSITY OF ALBERTA

ACE-NEURO
ALBERTA CANCER EXERCISE-NEURO

ACE-NEURO EXERCISE CLASS PROGRAMS

HOW CLASS PROGRAMS WORK

Each week in class, we will rotate between **Program A**, **Program B**, and **Program C**. We will provide modifications during class. Remember to listen to YOUR body first, our voices second. Please also remember to use the Rate of Perceived Effort (RPE) Scale to gauge how hard you are working in class. You can also find a number of exercise videos linked on our website: https://www.ucalgary.ca/healthandwellnesslab/resources

SAMPLE PROGRAM

PROGRAM A

CIRCUIT 1	
**EXERCISE**	**PROGRESSION/MODIFICATION**
(Ball) Wall Squat ***OR*** Body Weight Squat	• Progression: hold weight or increase range of motion<br>• Modification: sit to stand
Wall Angel	• Progression: increase wall angel range of motion and/or hold longer at end<br>• Modification: perform wall angel below 90 degrees, scapular retractions, or arm extensions (isometric)
Standing Hip Abduction	• Progression: monster walks or weighted/resisted hip abduction<br>• Modification: hold on and touch toe at end range of motion or seated clam shells
Cardio: High Knees	• Other cardio options or equipment
**CIRCUIT 2**	
Seated Band Row or Scapular Retractions	• Progression: increase resistance or perform either bent over row or one arm row w/ or w/out triceps kickback<br>• Modification: retractions and hold longer as tolerated
Standing Hamstring Curl	• Progression: use small ball, band, ankle weight, or pillow, from bodyweight; progress to single leg deadlift or glute busters<br>• Modification: modify to seated with band or isometric
Balance: Tandem Stance or Tandem Walk	• Progression: tandem walk<br>• Modification: feet unaligned or standing with support
Cardio: Bum Kicks	Other cardio options or equipment
**CIRCUIT 3**	
Chest Press (supine on floor)	• Progression: chest fly or increase weight or resistance<br>• Modification: perform seated or standing chest press or wall push ups
Dead Bug	• Progression: opposite arm/leg with pelvic tilt; or hold ball with movements<br>• Modification: pelvic tilt on floor or wall and progress hold of pelvic tilt
Bird Dog	• Progression: repeated with central touch for half the time or do on ball<br>• Modification: on the wall or floor with one limb at a time

SAMPLE PROGRAM

UNIVERSITY OF CALGARY
FACULTY OF KINESIOLOGY
Health and Wellness Lab

UNIVERSITY OF ALBERTA

ACE-NEURO
ALBERTA CANCER EXERCISE-NEURO

PROGRAM B

CIRCUIT 1	
Sumo Squats	• Progression: add weight/resistance and increase range of motion<br>• Modification: use chair or perform Sit to Stand, another bodyweight squat, or Wall Squat
Push-Ups	• Progression: wall, countertop, table, ottoman on, then toes<br>• Modification: chest press
Single Leg Balance	• Progression: A-Z balance; ball around waist or leg; Drinking Bird<br>• Modification: Tree Pose or holding onto support
Cardio: 2 in, 2 out or Low/High Jacks	Other cardio options or equipment
**CIRCUIT 2**	
Reverse Fly	• Progression: holding or increasing weight or resistance in standing hip hinge<br>• Modification: seated without resistance or just scapular restrictions
Calf Raises (dynamic balance)	• Progression: one leg, hold weight, or perform on stairs<br>• Modification: seated
Bicep Curl (round 1) /Triceps Kickback (round 2)	• Progression: increase weight or resistance, standing, bilateral triceps kickback<br>• Modification: no equipment – arm weight and/or one arm supported triceps kickback or standing press down
Cardio: Speed Skaters	Other cardio options or equipment
**CIRCUIT 3**	
Glute Bridges	• Progression: holding longer; walk outs; single-leg bridge; use stability ball<br>• Modification: glute squeeze (clench)
Plank Choice	• Variations: from wall, incline (e.g., counter; kneeling with hands on chair), or floor on knees to toes
Clamshells	• Progression: use a resistance/mini band around thighs, above knees, or add leg lift with knee extension<br>• Modification: seated on chair with band or use hands

SAMPLE PROGRAM

PROGRAM C

CIRCUIT 1	
Reverse Lunge	• Progression: side lunge, forward lunge, a three-way lunge, or step-ups<br>• Modification: quarter lunge, squat choice, or sit-to-stand bodyweight squat or sit to stand
Lat Pulldown	• Progression: increase resistance or perform A raises, lat pullover, or bodyweight pull-up<br>• Modification: lat pullbacks, scapular retractions, or arm extensions (isometric)
Monster Walk	• Progression: add resistance or side lunge<br>• Modification: seated clam shell or standing hip abduction
Cardio: Low/High Jumping Jacks ***OR*** Shadow Boxing	Other cardio options or equipment
**CIRCUIT 2**	
Row	• Progression: increase resistance, perform in standing/hip hinge position, or perform bilaterally<br>• Modification: one arm row or scapular retractions
Sumo Squats	• Progression: add resistance<br>• Modification: bodyweight option, ball wall squat, or seated leg extensions
Balance: Clock taps	• Progression: tap to 3,6,9 or 12<br>• Modification: hold a single leg stance with or without support
Cardio: Speed Skaters	Other cardio options or add equipment
**CIRCUIT 3**	
Chest Fly (supine)	• Progression: increase resistance or weight<br>• Modification: seated or standing option or push-up choice
Donkey Kick	• Progression: add resistance (dumbbell behind knee) or perform glute kickbacks with option of adding a resistance band above knees<br>• Modification: standing donkey kick, hip extension, or 4-point hip extension
Plank Choice	• Variations: from wall, incline (e.g., counter; kneeling with hands on chair), or floor on knees to toes

SAMPLE PROGRAM

UNIVERSITY OF CALGARY
FACULTY OF KINESIOLOGY
Health and Wellness Lab

UNIVERSITY OF ALBERTA

ACE-NEURO
ALBERTA CANCER EXERCISE-NEURO

Fatigue and Energy Thermometers

Fatigue

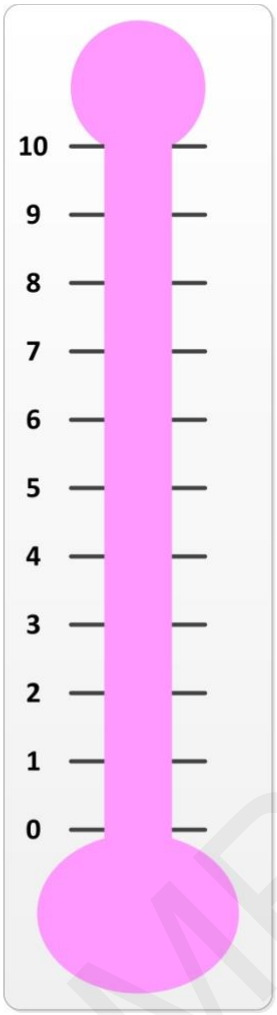

Completely Fatigued

Moderate Levels of Fatigue

No Fatigue at All

Energy

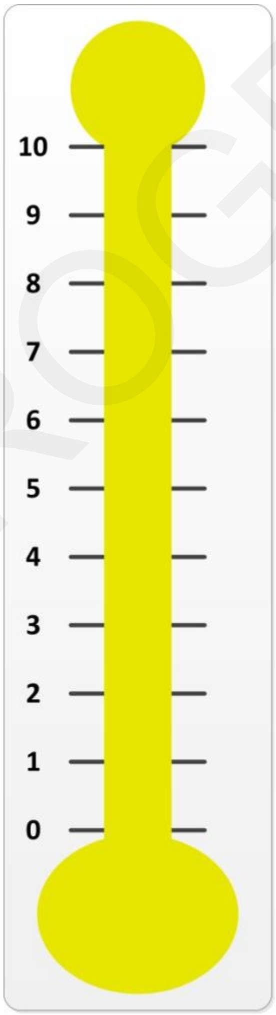

High Levels of Energy

Moderate Levels of Energy

No Energy at All

SAMPLE PROGRAM

UNIVERSITY OF CALGARY
FACULTY OF KINESIOLOGY
Health and Wellness Lab

UNIVERSITY OF ALBERTA

ACE-NEURO
ALBERTA CANCER EXERCISE-NEURO

How hard are you working?

Rating of Perceived Effort/Exertion Scale – Adapted from Borg 1998

0 Nothing at all

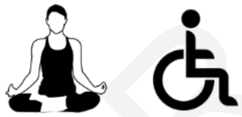

0.5 Very, very light

like a person walking slowly at their own pace

1 Very light

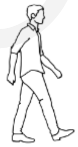

2 Light

3 Moderate — not especially hard - no problem continuing

4 Somewhat hard

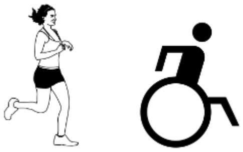

5 Hard

heavy exercise - it feels hard - no problem continuing

6 Hard

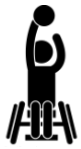

7 Very hard

strenuous exercise - person has to push themselves

8 Very hard

9 Very hard

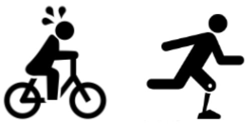

10 Very, very hard (maximal)

extremely strenuous exercise - the hardest you have ever experienced

SAMPLE PROGRAM
