# Supplementary material for: The feasibility of a multi-site, clinic-supported, and tailored neuro-oncology exercise program
Source: Neurooncol Pract. 2024 Oct 10;12(1):131–42. doi: 10.1093/nop/npae093 (PMC11798609; doi:10.1093/nop/npae093)
Supplement: npae093_suppl_Supplementary_Appendix_B [file npae093_suppl_supplementary_appendix_b.pdf]

ACE-Neuro: Tailored Exercise Program (Initial Program)

Name: First Name, Last Name
Date: MM-DD-YYYY

UNIVERSITY OF CALGARY
FACULTY OF KINESIOLOGY
Health and Wellness Lab

ACE-NEURO
ALBERTA CANCER EXERCISE-NEURO

Total Time to Complete: 15-60 min.

Begin with a 5-7+ minute warm-up. End with a 7-10-minute+ stretch.

Complete **1-3 sets** *(# of rounds)* of the circuits below. Complete **45-60 seconds** per exercise.
You can complete both circuits at a time or one circuit one day, the other another day.

On the days you feel more **energized**, complete 2-3 rounds and 60 seconds per exercise at an RPE (rating of perceived effort) of 3-6.
On the days you feel more **fatigued**, complete 1 round and 45 seconds per exercise OR choose 1-3 exercises to complete (instead of the full program) at an RPE of 1-3.

Use the Rating of Perceived Effort/Exertion Scale and Fatigue and Energy Scales on the last pages of your program to monitor your fatigue and energy pre and post-exercise and effort during exercise.

EXERCISE	INSTRUCTIONS	DEMONSTRATION
**CIRCUIT #1: STANDING AND CARDIO EXERCISES**		
**1. SQUAT**	**This exercise primarily works your quads (thigh muscles) and glute (buttocks muscles)**<br>→Start with your feet about shoulders-width apart.<br>→Send your hips and buttocks as far down and back as feels comfortable, bending at the knees.<br>→Ensure the movement comes from your hips.<br>→Return back to standing.<br>→To make it **EASIER**: decrease the distance you squat down.<br>→To make it **HARDER**: add resistance (e.g., hold a dumbbell, water bottle) and/or squat lower.<br>→Altnerative exercise options: sit-to-stand, leg extension.	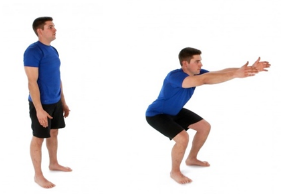
**2. CHEST PRESS**	**This exercise primarily works your pectoral (chest muscles)**<br>→You can perform this exercise standing, seated, or from a bench or floor.<br>→If using a band, loop the band around your back and keep under your armpits.<br>→Roll your shoulders down and back from your ears to set your posture.<br>→Start with your elbows bent, palms facing downards, and hands at chest-level.<br>→Press your hands forwards until your arms are almost straight (maintain slight bend in elbows).<br>→To make it **EASIER**: decrease or remove resistance.<br>→To make it **HARDER**: increase resistance (e.g., band, dumbbells, etc.).<br>→Altnerative exercise options: chest press on cable machine, push-ups.	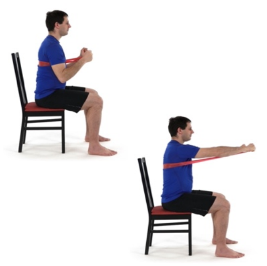

SAMPLE PROGRAM

**3. SINGLE LEG FORWARD BEND**	**This exercise primarily works your hamstring (back of legs) and glute (buttocks musles), as well as balance and flexibility**<br>→Start with your feet about hip distance apart.<br>→Set your posture by straightening your back and engaging your core.<br>→Lift one leg from the floor.<br>→Keeping your back straight and chest lifted, hinge at your hips to bend forwards.<br>→Maintain a slight bend in the standing leg and keep your hips and chest parallel to the direction you are facing.<br>→Bend as far as your hamstrings will allow.<br>→Return to the standing position by squeezing your buttocks muscles and repeat.<br>→To make it **EASIER**: perform with both feet planted on the floor or a single leg stance.<br>→To make it **HARDER**: hold the bend for longer or add resistance (e.g., hold a dumbbell, water bottle).<br>→Altnerative exercise options: standing hamstring curl.<br>.	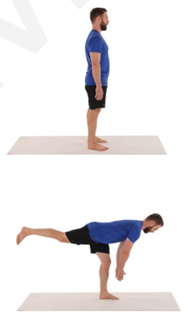
**4. LAT PULLDOWN**	**This exercise primarily works your latissimus dorsi (back muscles) and arm muscles**<br>→You can perform this exercise standing or seated.<br>→If using a band, anchor it above your head or hold the band about shoulders-width apart at or above eyebrow height.<br>→Drawing your shoulder blades together and toward the ground, pull the band apart and down to lower the arms on each side.<br>→Keep the band in FRONT of your body.<br>→To make it **EASIER**: perform without a band or try one arm at a time.<br>→To make it **HARDER**: use a harder band and/or hold the bottom of your movement for longer.<br>→Altnerative exercise options: wall angel, reverse fly, row.	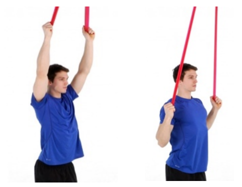
**5. SIDE LUNGE**	**This exercise primarily works your quads (thighs), glutes (buttocks), and hip muscles**<br>→Start standing with your feet hip distance apart.<br>→Take a sizeable step to one side.<br>→Send your hips and buttocks back.<br>→Push through your heels to return to the starting position.<br>→Repeat on the other side.<br>→To make it **EASIER**: lunge side to side without lifting the feet and/or decrease the distance your hips go back.<br>→To make it **HARDER**: add resistance (e.g., hold a dumbbell, water bottle) and/or lunge lower.<br>→Altnerative exercise options: side leg kick.	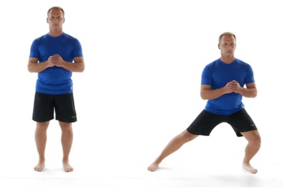

SAMPLE PROGRAM

**6. BICEP CURL TO SHOULDER PRESS**	**This exercise primarily works your biceps and shoulder muscles**<br>→Stand with dumbbells (or water bottles, cans of soup) in your hands or standing on a band.<br>→Curl your dumbbell/band up with palms facing forward.<br>→At the top of the curl, press the dumbbells overhead.<br>→Keep your back straight and core engaged, preventing your back from arching.<br>→Slowly lower and repeat.<br>→To make it **EASIER**: decrease or remove resistance and/or perform the curl without the press.<br>→To make it **HARDER**: increase resistance.<br>→Altnerative exercise options: bicep curl to lateral raise.	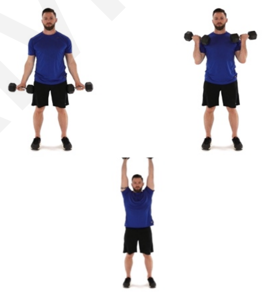
**7. CARDIO**	**Cardio exercise primarily works your heart muscle and cardiovascular system**<br>→Choose your favourite bodyweight or machine cardio exercise to perform.<br>→Some ideas include: bum kicks, high knees, speed skaters, jumping or low jacks, fast feet, two-in, two-out.<br>→To make it **EASIER**: perform low-impact and slower cardio exercises.<br>→To make it **HARDER**: perform higher impact, more complex, or faster cardio exercises.<br>→Alternative exercise options: rest & re-hydrate **OR** perform cardio exercise on a separate day.	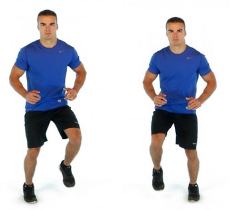
**CIRCUIT #2: CORE/ FLOOR EXERCISES**		
**1. Is Ys Ts**	**This exercise primarily works the mobility of your chet, shoulders, and back**<br>→Complete this exercise on your back or standing.<br>→Reach one or two arms at a time overhead and then draw the shape of an I.<br>→Reset and repeat by drawing the shape of an Y.<br>→Reset and repeat by drawing the shape of a T.<br>→Alternative exercise options: angels.	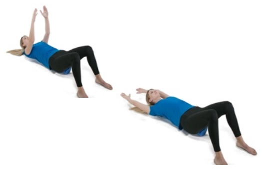
**2. CORE ENGAGEMENT**	**This exercise primarily works your transversus abdominus (deep core muscle)**<br>→Lay on your back on a firm and flat surface with your knees bent and your feet flat.<br>→You can also perform this exercise standing.<br>→Place your hands just above your hip bones and slightly toward the centre of your belly to monitor the contraction of the transversus abdominis muscle.<br>→Find your neutral spine position by slowly moving your hips to press your back into the floor (or wall if standing), then slowly moving your hips to lift your back away from the floor/wall. Slowly move between these two positions to find your most comfortable and neutral spine position.<br>→Keep your neutral spine position throughout the exercise.<br>→Gently pull your belly button toward your spine. You should feel a slow and deep tension under your fingers without any superficial abdominals activation or any movement of the pelvis.<br>→Hold the contraction for a few seconds before relaxing.<br>→Repeat.	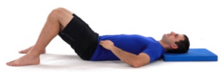<br>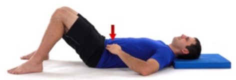

SAMPLE PROGRAM

3. HEEL DROPS	This exercise primarily works your transversus abdominus (deep core muscle)<br>→Lie on your back with your knees bent and your back in a neutral position.<br>→Engage your core.<br>→Maintain a steady abdominal breathing while you lift one leg up to 90 degrees at the hip and keeping the knee bent.<br>→Keep your back and pelvis completely still.<br>→Return slowly to the initial position and repeat with the other leg.<br>→To make this EASIER: core engagement and deep breathing.<br>→To make this HARDER: lift both legs up to 90 degrees and slowly drop one leg at a time to the floor.<br>→Alternative exercise options: dead bug.	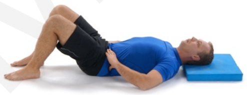<br>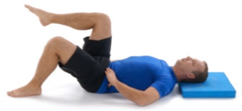
4. GLUTE BRIDGE	This exercise primarily works your glute (buttocks) and hamstring (back of leg) muscles<br>→Lie on your back with your knees bent.<br>→Contract your buttocks to lift your hips off the ground until the trunk of your body is aligned with your legs.<br>→Slowly return to the initial position and repeat.<br>→To make this HARDER: hold at the top of the bridge for longer, add a single leg kick-out, perform one legged, squeeze a ball between your knees during the bridge, or add a pulse at the top of your bridge (where knees pulse outwards).<br>→Alternative exercise options: glute kickbacks (standing), hip thrusts.	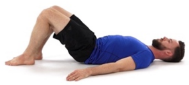<br>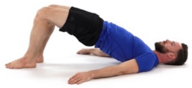
5. CLAM SHELL	This exercise primarily works your gluteus medius (side buttocks), hips, and leg muscles<br>→Lie on your side with both legs slightly bent.<br>→Stack your hips on top of each other and shoulders on top of each other.<br>→Lift your top leg whilst keeping your knee bent and feet together.<br>→Return to the initial position and repeat.<br>→To make this HARDER: hold at the top of your movement for longer or add a resistance band above your knees.<br>→Alternative exercise options: side leg kick/hip abduction.	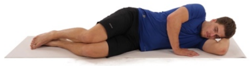<br>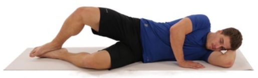
6. BIRD DOG	This exercise primarily works your core – lower abdominal muscles, back muscles, and buttocks (glute muscles)<br>→Get on your hands and knees (four-point/ tabletop position) with your knees directly under your hips and your hands directly under your shoulders.<br>→Your back is in a neutral position and your chin is be tucked in.<br>→Engage your core by tightening your lower abdominal muscles, lumbar muscles, and pelvic floor muscles.<br>→Lift one arm or one leg away from the body, without allowing the trunk or pelvis to move or rotate.<br>→If you feel stable, lift the opposite arm or leg away from the body as well.<br>→Imagine grabbing something far away in front of you with your hand and touching an imaginary wall far behind you with your foot.<br>→Lower your leg and arm back to the floor and repeat with the other leg and the opposite arm.<br>→To make it EASIER: perform from standing/using a chair or only lift your leg or arm instead of both.<br>→To make it HARDER: hold for longer or try balancing a light object on your back.<br>→Alternative exercise options: plank, bear plank.	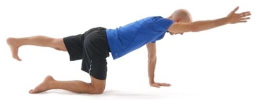<br>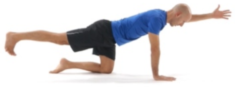

SAMPLE PROGRAM

How hard are you working?

Rating of Perceived Effort/Exertion Scale – Adapted from Borg 1998

0 Nothing at all

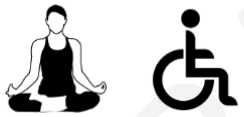

0.5 Very, very light

like a person walking slowly at their own pace

1 Very light

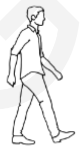

2 Light

3 Moderate — not especially hard - no problem continuing

4 Somewhat hard

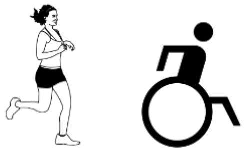

5 Hard

heavy exercise - it feels hard - no problem continuing

6 Hard

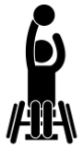

7 Very hard

strenuous exercise - person has to push themselves

8 Very hard

9 Very hard

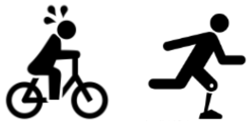

10 Very, very hard (maximal)

extremely strenuous exercise - the hardest you have ever experienced

SAMPLE PROGRAM

Fatigue and Energy Thermometers

Fatigue

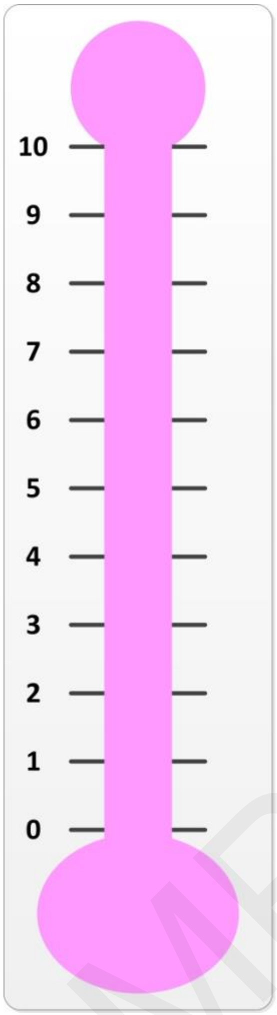

Completely Fatigued

Moderate Levels of Fatigue

No Fatigue at All

Energy

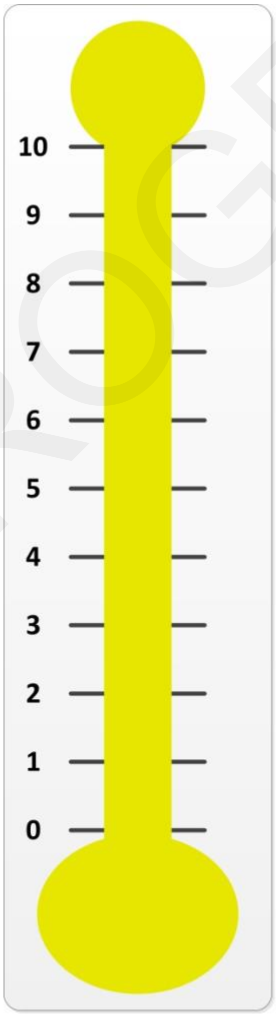

High Levels of Energy

Moderate Levels of Energy

No Energy at All

SAMPLE PROGRAM
